# Immersive Virtual Reality as an Adjunctive Non-opioid Analgesic for Pre-dominantly Latin American Children With Large Severe Burn Wounds During Burn Wound Cleaning in the Intensive Care Unit: A Pilot Study

**DOI:** 10.3389/fnhum.2019.00262

**Published:** 2019-08-08

**Authors:** Hunter G. Hoffman, Robert A. Rodriguez, Miriam Gonzalez, Mary Bernardy, Raquel Peña, Wanda Beck, David R. Patterson, Walter J. Meyer

**Affiliations:** ^1^Department of Mechanical Engineering, College of Engineering, University of Washington, Seattle, WA, United States; ^2^Psychiatry and Behavioral Sciences, University of Texas Medical Branch at Galveston, Galveston, TX, United States; ^3^Shriners Hospitals for Children, Galveston, TX, United States; ^4^Department of Rehabilitation Medicine, University of Washington, Seattle, WA, United States

**Keywords:** virtual reality, pain, pediatric burn injuries, analgesia, critical care, burn, opioid, developing countries

## Abstract

**Background/Aim:** Using a within-subjects, within-wound care design, this pilot study tested for the first time, whether immersive virtual reality (VR) can serve as an adjunctive non-opioid analgesic for children with large severe burn wounds during burn wound cleaning in the ICU, in a regional burn center in the United States, between 2014–2016.

**Methods:** Participants included 48 children from 6 years old to 17 years of age with >10% TBSA burn injuries reporting moderate or higher worst pain during no VR on Day 1. Forty-four of the 48 children were from developing Latin American countries. Patients played adjunctive SnowWorld, an interactive 3D snowy canyon in virtual reality during some portions of wound care, vs. No VR during comparable portions of the same wound care session (initial treatment condition randomized). Using Graphic Rating scales, children's worst pain ratings during “No VR” (treatment as usual pain medications) vs. their worst pain during “Yes VR” was measured during at least 1 day of wound care, and was measured for up to 10 study days the patient used VR.

**Results:** VR significantly reduced children's “worst pain” ratings during burn wound cleaning procedures in the ICU on Day 1. Worst pain during No VR = 8.52 (SD = 1.75) vs. during Yes VR = 5.10 (SD = 3.27), *t*_(47)_ = 7.11, *p* < 0.001, SD = 3.33, CI = 2.45–4.38, Cohen's d = 1.03 (indicating large effect size). Patients continued to report the predicted pattern of lower pain and more fun during VR, during multiple sessions.

**Conclusion:** Immersive virtual reality can help reduce the pain of children with large severe burn wounds during burn wound cleaning in the Intensive Care Unit. Additional research and development is recommended.

## Introduction

Acute pain is a frequent medical problem world wide, but children with large severe burn injuries (e.g., 40% TBSA) experience some of the most painful procedures in medicine. During the course of their weeks in the hospital burn center's intensive care unit, children with large severe burns must have their wounds cleaned/scrubbed frequently to prevent infection and speed up healing. Opioid analgesics are widely regarded as effective and essential tools for acute pain management (Malchow and Black, 2008; Vijayan, 2011; McIntyre et al., 2016; Ballantyne, 2018; Krane, 2019). According to Berterame et al. (2016, p. 1664) “*In developing countries, access to opioids is very limited. In 2009, more than 90% of worldwide use of opioid analgesics occurred in the USA, Canada, Australia, New Zealand, and several European countries. Use in that year was deemed low in 21 countries and very low in more than 100.”* Patients in Latin American often have limited access to opioids for pain control (used for both analgesia and anesthesia). Yet even in the U.S.A., there are currently shortages of pharmaceutical medical opioid analgesics needed for acute pain control during medical procedures (Davis et al., 2018). And because of a large increase in opioid related overdose deaths unrelated to burn patients (Chen et al., 2019), there is growing political and legal pressure to further reduce reliance on opioids for pain control in the U.S.A.

For patients treated with opioid pain medications (e.g., patients treated in regional hospital burn centers in the United States), opioid side effects (Dunwoody and Jungquist, 2018) limit dose levels, limiting analgesic effectiveness (Cherny et al., 2001; Malchow and Black, 2008; Clark et al., 2017; Ballantyne, 2018). And opioid tolerance/habituation is a challenge for patients with large severe burns (Bittner et al., 2015), who typically receive the same painful procedures over and over, several times per week, often daily, during several weeks of hospitalization. Excessive pain and/or repeated high opioid doses can pathologically alter the patients pain perception system, disrupting the patient's natural endogenous opioid analgesia system (Schwaller and Fitzgerald, 2014; Ballantyne, 2018; Chambers, 2018), and can increase patient's risk of developing chronic pain, anxiety disorders, and/or Post-Traumatic Stress Disorder (McGhee et al., 2011; Rosenberg et al., 2015, 2018; Pardesi and Fuzaylov, 2017; Peña et al., 2017).

Psychological factors such as fear, anxiety, and depression can increase or amplify how much pain patients subjectively experience during painful medical procedures (Hemington et al., 2017; Nitzan et al., 2019), making pain management even more challenging. What people are thinking about during wound care, and where patients direct their attention during medical procedures can influence pain intensity (Melzack and Wall, 1965). For example, if patients predict wound care is going to be painful, that can make their pain worse. According to Fields (2018, p. S8) “…expectation of pain becomes a self-fulfilling prophecy through top down amplification of the pain signal,” and memories of previous painful procedures can also increase pain intensity (Noel et al., 2015).

Fortunately, just as psychological factors can make pain worse, psychological treatments can help reduce acute pain during medical procedures. For example, distraction techniques (e.g., music) are widely used in clinical practice, and can be used in addition to traditional pain medications to help control pain during burn wound care. Some studies show strong benefits of music therapy during burn wound care in patients (e.g., Rohilla et al., 2018). But in other studies the benefits of listening to music during burn wound care had small effect sizes and/or non-significant results (Fratianne et al., 2001; Bellieni et al., 2013; van der Heijden et al., 2018), and/or involved patients with small burn wounds (e.g., 5% TBSA, Hsu et al., 2016).

For the extreme pain levels experienced by children with large severe burn wounds during burn wound debridement in the intensive care unit, creating stronger non-pharmacologic pain control techniques is a national and international priority (Keefe et al., 2018).

Immersive virtual reality is a promising new non-opioid psychological pain distraction technique. There is growing evidence that adjunctive immersive virtual reality distraction can significantly reduce how much pain patients experience during a growing number of different painful medical procedures e.g., during urological endoscopies, physical therapy after surgery for cerebral palsy, venipuncture for onco-therapy, and pediatric dental procedures (Hoffman et al., 2011; Garrett et al., 2014; Scheffler et al., 2017; Atzori et al., 2018a,b; Indovina et al., 2018; Honzel et al., 2019).

Brain scan studies provide converging evidence that VR reduces acute pain. Using neuroimaging assessments, a laboratory functional magnetic resonance imaging study found that in addition to reducing subjective pain ratings, VR reduced pain-related brain activity (Hoffman et al., 2004b). In a second fMRI brain scan study, the amount of pain reduction from VR alone was comparable to the amount of pain reduction from a moderate dose of hydromorphone, and “VR + opioids” combined resulted in the largest pain reductions (Hoffman et al., 2007).

The logic for why VR would reduce pain is based on an attentional mechanism (Hoffman, 1998; Hoffman et al., 2000, 2006). The essence of immersive virtual reality analgesia is the patient's illusion of going to a different place, the subjective experience of “feeling present” in the computer generated world, as if the virtual reality world is a place they are visiting (Slater and Wilbur, 1997). Human brains are limited in how much information they can process (Kahneman, 1973). Pain requires attention. Researchers argue that the illusion of “being there” in virtual reality is unusually attention grabbing, reducing the amount of attentional resources the patient's brain has available for pain perception (Hoffman, 1998; Hoffman et al., 2000, 2003;Hoffman et al., 2004a).

According to a gate control theory explanation of psychological analgesia (Melzack and Wall, 1965, p. 978), “…psychological factors such as past experience, attention, and emotion can influence pain response and perception….” Melzack and Wall proposed that the brain may inhibit nociceptive signals.

Regardless of the mechanism, several small clinical studies have shown encouraging preliminary evidence that adjunctive VR can help reduce pain during burn wound care in adults (Hoffman et al., 2004a, 2011; van Twillert et al., 2007; Maani et al., 2011a,b; McSherry et al., 2018), and in children with small burns, (Hoffman, 1998; Hoffman et al., 2000; Faber et al., 2013; Jeffs et al., 2014; Khadra et al., 2018). There is also preliminary evidence that VR is more effective than conventional distractions such as video games or movies. In the first study to report using immersive virtual reality for pain control during a medical procedure, two adolescent boys with large burn injuries underwent staple removals from healing burn skin grafts during immersive VR vs. while playing a Nintendo video game (no VR). Both patients reported large reductions in pain during staple removal during immersive virtual reality compared to their pain during staple removal while playing the (no VR) traditional Mario Kart Nintendo video game (Hoffman et al., 2000) during the same wound care session. More recently, in a study by Jeffs et al. (2014) adolescent burn patients with small burns (5% TBSA) treated in an outpatient clinic reported significantly lower pain during virtual reality compared to a group that watched a movie during wound cleaning.

There are a number of barriers to using VR in the ICU tubroom. The patients in the current study had a burn size of 40% Total Body Surface Area (TBSA). As is often the case for patients with such unusually large severe burn injuries, most of the burn patients in our study had head and face burns, preventing them from wearing a conventional commercially available head mounted VR helmet. Furthermore, even when treated with powerful pain medications, pain during burn wound care procedures in the ICU hydrotank is often “severe to excruciating,” which may make it harder for children to concentrate enough to play in VR during wound care. In theory, pain may become so attention grabbing that psychological distraction techniques cannot compete with pain for the patient's limited attention (Eccleston and Crombez, 1999; Eccleston, 2001). In other words, some patients may not benefit from VR if their acute procedural pain becomes too intense. Similarly, traditional distraction may fail if patients feel threatened during the wound care (McCaul and Malott, 1984; Crombez et al., 1998). High catastrophizers (people who have unusually negative emotions and pessimistic beliefs about their ability to deal with the upcoming pain) may have difficulty disengaging attention from pain information (Verhoeven et al., 2012; Van Loey et al., 2018).

To address these challenges, using a custom water-friendly VR system, the current pilot study tests for the first time, whether adjunctive virtual reality can reduce the acute procedural pain of children with large severe burn injuries during burn wound debridement/cleaning in the pediatric intensive care unit, in an understudied patient population, critically injured pediatric patients.

We hypothesize that compared to standard of care (standard pain medications + No VR), during adjunctive Yes VR, children will report significant reductions in worst pain ratings. Our secondary hypothesis is that during VR, children will report significant reductions in pain unpleasantness, and will spend less time thinking about pain during burn wound debridement in the ICU hydrotank. We further hypothesize that VR will increase how much fun patients have during wound care, and that patients will be more satisfied with their pain management during VR.

## Materials and Methods

This research was conducted between Jan 2014 and Dec 2016, in accordance with the Declaration of the World Medical Association (www.wma.net). The studies were approved by the IRB from UTMB, and all participants and their parents/legal guardians provided written informed consent/assent in accordance with the Declaration of Helsinki.

Most of the children in the current study were transported from Latin America to Shriners Hospitals for Children in Galveston Texas, U.S.A., where they were hospitalized, treated, and returned to their country of origin, post-discharge.

### Inclusion Criteria

Children were included in the study if they were (1) compliant and able to complete subjective evaluations, (2) had no history of previous psychiatric (DSM-III-R Axis I) disorder(s), (3) were not demonstrating delirium, psychosis, or any form of organic brain disorder, (4) were able to communicate verbally in English or Spanish, and (5) had moderate or higher worst pain during no VR on Day 1, (6) were admitted to Shriners Hospitals for Children: Galveston Texas/University of Texas Medical Branch.

Children were excluded from the study if (1) they had a burn size <10% TBSA, (2) they were not capable of completing the study measures, (3) if no wound cleaning sessions were required, (4) if they had a history of previous psychiatric (DSM-III-R Axis I) disorder(s), (5) if they were demonstrating delirium, psychosis, or organic brain disorder, (6) if the child was unable to communicate verbally in English or Spanish, (7) if they had a history of significant cardiac, endocrine, neurologic, metabolic, respiratory, gastrointestinal, or genitourinary impairment, (8) if they were receiving prophylaxis for alcohol or drug withdrawal, (9) if they had a developmental disability, (10) if they were younger than 6 years old, (11) if they were older than 17 years old, or (12) if they had burns of eyes, eyelids, or face so severe the burns precluded the use of VR equipment, (13) or if patients reported having a previous history of severe motion sickness.

### Equipment

**Figure 1:**
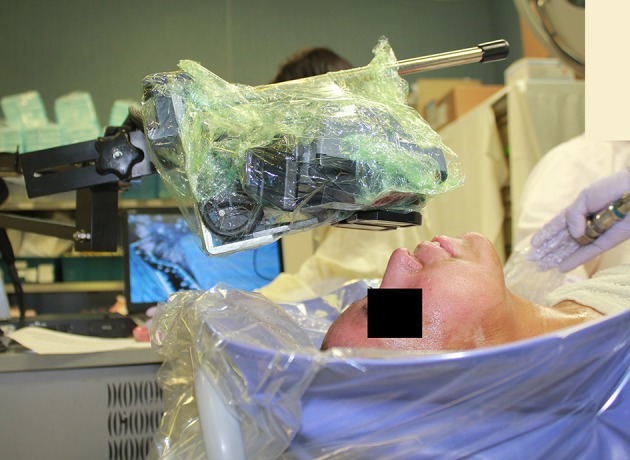
A patient playing SnowWorld during burn wound debridement in the ICU tankroom. Photo and copyright Hunter Hoffman, www.vrpain.com.

**Figure 2:**
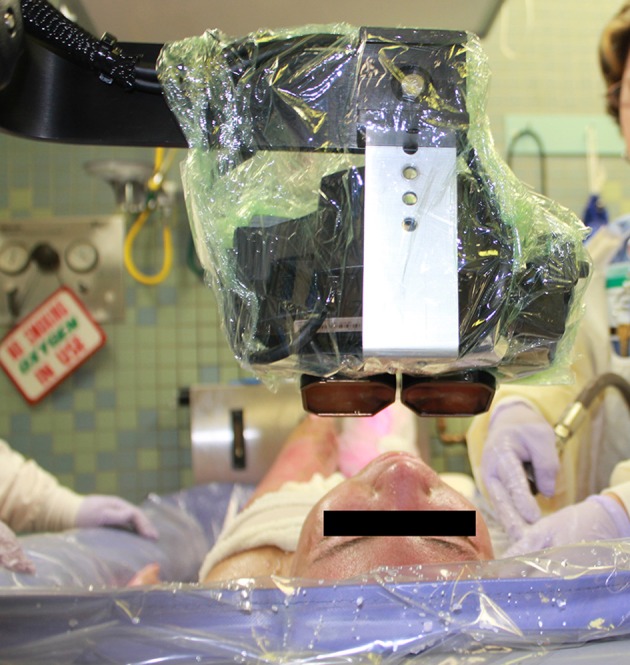
A patient looking into VR goggles during burn wound debridement in the ICU tank room. Photo and copyright Hunter Hoffman, www.vrpain.com.

The current study introduced for the first time, a new portable water-friendly VR system customized for the unique needs of pediatric patients with large severe burn injuries during wound care in the intensive care unit hydrotank. As shown in Figure 1, a custom robot-like articulated arm goggle holder was used in the current study to hold a pair of VR goggles near the patient's eyes, so patients did not have to wear a VR helmet on their head. This “Magula arm” robot-like goggle holder minimized or ideally eliminated contact between the patient and the VR goggles. The VR goggles largely blocked the patient's view of the Intensive Care Unit hydrotank room. The goggles were MX90 VR goggles, from NVIS.com, with 90 degrees field of view diagonal, per eye, and 1,280 × 1,024 pixels resolution per eye. All of the VR equipment in the current study was battery powered. A battery powered laptop and battery powered audio-visual unit were used with the MX90 VR goggles. The 90 degrees diagonal field of view goggles increased the amount of peripheral vision stimulated. During the VR condition, patients were encouraged to interact with the virtual environment via a wireless computer mouse. Stereo speakers helped isolate patients from hearing hospital sounds. The custom robot-like articulated arm goggle holder was securely mounted to the frame of the Anthro medical cart. The VR goggles orientation could be adjusted and locked into position for a patient who was sitting up during wound care, or the goggles could be rotated and locked into position for a patient who was lying on their backs during wound care (see Figure 2). The goggles stayed in one position, and the patient used their wireless mouse to look around, aim and shoot snowballs in SnowWorld (mouse-tracking instead of head tracking).

The portable robot-like arm goggle holder was designed by Hoffman and Magula and built by Jeff Magula, an advanced instrument maker at the University of Washington in Seattle. Once finished, the water-friendly VR system was then safety inspected by Clinical Engineering at the University of Washington, and was inspected again by Clinical Engineering at Shriners Hospitals for Children. The equipment was also approved for use in the Intensive Care Unit and the equipment cleanliness was monitored by infection control at Shriners Hospitals for Children. After each use, the VR cart/portable VR system was returned to the Psychology Department at Shriners Galveston, where it was plugged in to recharge the batteries after each use. As shown in Figure 2, the goggles were partially covered with disposable plastic, which was discarded after each use. The equipment was systematically disinfected after each use using chemical disinfectants, and was periodically supercleaned using ultraviolet radiation (using a portable UV lamp wand, UV protective glasses, while wearing latex gloves). For example, the UVC Blade Handheld Germicidal Fixtures by American Ultraviolet. The VR system was periodically tested for pathogens, using swabs that were then analyzed by Shriners infection control, to test for the presence of bacteria. Culture samples (swabs) were sent to the microbiology laboratory at Shriners hospital in Galveston for immediate analysis. The post-cleaning tests all came back as “safe” (no pathogens). There was no significant problem with infection, using the current VR system, which minimized or eliminated physical contact between the patient and the VR goggles.

## Measures

After each wound care session, subjects received the following instructions once prior to answering each of five separate questions. “Please indicate how you felt during wound care today by making a mark anywhere on the line. Your response doesn't have to be a whole number.”

For the primary dependent measure, using Graphic Rating Scales (GRS), after the wound care session, patients answered the following GRS ratings. Pain was measured using Graphic Rating Scales (GRS) (Jensen and Karoly, 2001; Jensen, 2003). In the current study, the GRS tool was used to assess three reports of the pain experience (“worst pain,” “pain unpleasantness,” and “time spent thinking about pain”) that correspond to three separable components of the pain experience; sensory pain, affective pain, and cognitive pain, respectively. The GRS is a 10-unit horizontal line labeled with number and word descriptors. Descriptor labels were associated with each mark to help the respondent rate pain magnitude in each domain. For worst pain, the GRS descriptors were *no pain at all, mild pain, moderate pain, severe pain*, and *excruciating pain*. For pain unpleasantness, the GRS descriptors were *not unpleasant at all, mildly unpleasant, moderately unpleasant, severely unpleasant*, and *excruciatingly unpleasant*. For time spent thinking about pain, the GRS descriptors were *none of the time, some of the time, half of the time, most of the time, all of the time*.

The Graphic Rating Scale has previously been used to assess pain intensity in children eight and older and has been documented to be the preferred report method for young children (Tesler et al., 1991). The GRS is more sensitive than simple descriptive pain scales and patients can easily answer these pain ratings despite having no previous experience. Visual Analog Scales have been validated for use in children aged 7 and higher (Bringuier et al., 2009).

A single rating “to what extent did you feel like you ‘went into’ the virtual world,” adapted from Slater et al. (1994) was also used in the present study to assess user presence in the virtual world. Descriptor labels were *I did not feel like I went inside at all, mild sense of going inside, moderate sense of going inside, strong sense of going inside, I went completely inside the computer generated world*. Hendrix and Barfield (1995) showed the reliability of a similar VR presence rating. The measure's ability to detect treatment effects (Hoffman et al., 2004c) is preliminary evidence of our VR presence measure's validity. Patients also rated how real the objects seemed in virtual reality, descriptors were *completely fake, somewhat real, moderately real, very real, indistinguishable from a real object*. Patients rated how satisfied they were with their pain management during No VR vs. during VR, with descriptors *completely unsatisfied, mostly unsatisfied, half satisfied, mostly satisfied, completely satisfied*, and patients rated nausea as a result of VR, using a graphic rating scale with descriptors *no nausea at all, mild nausea, moderate nausea, severe nausea, vomit*. All text was translated into Spanish for Spanish speaking participants using an official translator (90% of the participants in this study were Spanish only speaking). To assess whether patients in the upper quartile on catastrophizing showed pain reduction during immersive Virtual Reality, we administered the Pain Catastrophizing Scale for Children (PSC-C) (Sullivan et al., 1995; Crombez et al., 2003). The PCS total score is calculated by summing the 13-item responses, and provides a good index of the catastrophizing construct through the inclusion of highly correlated subscales of helplessness, rumination, and magnification. Higher scores on the PCS-C are indicative of greater pain-related catastrophizing. The PCS-C has been validated for use with children (Crombez et al., 2003).

### Experimental Design

There is high variability in the analgesic effectiveness of any given dose of pharmacologic analgesia from one burn wound care session to the next (Khadra et al., 2018). And furthermore, pain medication dose levels can also vary from day to day. For these reasons, in the current preliminary study, a statistically powerful within-subjects, within-wound care design was used (Maani et al., 2011a). During VR, patients played SnowWorld, an interactive 3D snowy canyon in virtual reality during some portions of wound care, vs. No VR during comparable portions of the same wound care session. Childrens' worst pain during “No VR” (treatment as usual pain medications) vs. their worst pain during “Yes VR” was measured during at least 1 day of wound care, and was measured for up to 10 study days the patient used VR. Initial treatment order was randomized using blocked randomization, based on random number sequences generated using www.random.org. All patients received their usual pain medications on all study days, i.e., VR was always used adjunctively, in addition to usual traditional pain medications.

During wound care, the nurses cut off and removed the patient's gauze bandages, and began cleaning the patients burn wounds, using warm wet washcloths and a hand held warm water shower hose to scrub and rinse away dead tissue and debris out of the burn wound. During wound debridement, patients received No VR and Yes VR during approximately equally painful portions of the same wound care session. The patient began receiving wound care for 5 min with Yes VR vs. 5 min with No VR, Yes VR for five more minutes, etc. repeatedly alternating between No VR and Yes VR every 5 min. Whether patients received Yes VR or No VR during the first 5 min treatment segment was randomized (blocked randomization using a random sequence generated at random.org). During the portions of their burn wound care that they received VR, the research staff positioned the VR goggles weightlessly near the patient's eyes, with little or no physical contact between the VR goggles and the patient, using a robot-like-arm goggle holder (Maani et al., 2008). The patient looked into the VR goggles, and interacted with the virtual reality world.

**Figure 3:**
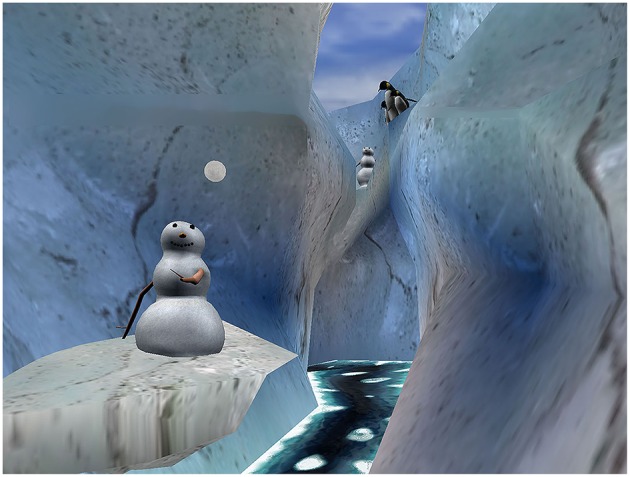
SnowWorld. An icy 3D canyon in virtual reality. Image by Ari Hollander and Howard Rose, copyright Hunter Hoffman, www.vrpain.com.

All patients used SnowWorld (see Figure 3) during all VR sessions. SnowWorld is a non-profit VR world specifically designed for pain distraction of immobilized severe burn patients, including children. SnowWorld is designed to give burn patients the illusion of going inside a snowy 3D canyon (Hoffman et al., 2001, 2004b,c; see also Bloemink et al., 2006, p. 104–106). In SnowWorld (www.vrpain.com), patients interacted with snowmen, igloos, penguins, wooly mammoths, and flying fish by throwing snowballs, using a wireless computer mouse to aim and trigger snowballs while keeping their heads and bodies motionless. During VR, patients heard music (e.g., Paul Simon's song Graceland, and several Spanish songs), and 3D sound effects e.g., ice breaking when a snowball hits a snowman. Mammoths trumpeted angrily when pelted.

After the wound care session was over, patients briefly rated how much pain they had experienced during No VR vs. during Yes VR using graphic rating scales. The patient's burns were rebandaged, the patient was wheeled back to their hospital room and returned to their hospital beds, and the research staff thoroughly cleaned and disinfected the VR equipment.

### Statistical Analyses

IBM SPSS (2018) statistical analyses of the primary and secondary hypotheses involved an apriori two-tailed within-subjects paired *t*-test, with alpha = 0.05.

## Results

Patients participated between January 2014 and December 2016. Out of the 62 patients initially screened, 48 pediatric patients met our apriori inclusion criterion of having a moderate or higher “worst pain” rating during No VR on Day 1 (33 hispanic males children, 11 hispanic female children from developing Latin American countries, and also three non-hispanic female children and one non-hispanic male from the United States). The mean size of the patient's severe burn injuries was 40 percent Total Body Surface Area (TBSA) burned, 28% third degree burns. Patients' ages ranged from 6 to 17 years of age at time of enrollment (Mean age was 12 years old). Seventy-seven percent of the patients had hand burns, 85% had arm burns, 44% had foot burns, 79% had leg burns, 71% had neck/head burns, 79% had trunk/torso burns, and 23% had groin burns. Regarding the (sometimes overlapping) etiology of their burns, 81% had burns involving flame, 6% scalds, 25% electrical, and zero patients had chemical burns.

### Test of Our Primary Hypothesis

**Figure 4:**
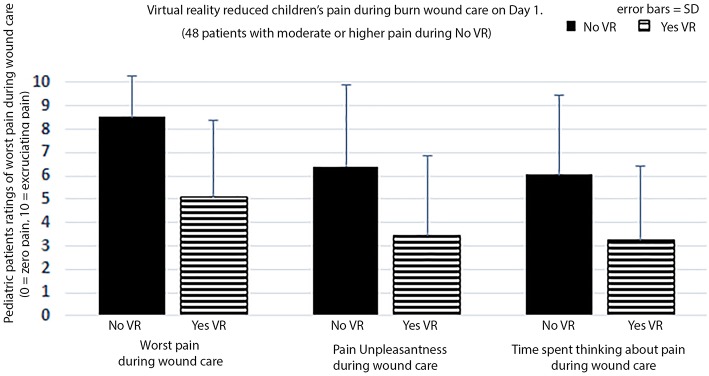
Patients with moderate or higher pain ratings during wound care on Day 1.

The patients GRS pain ratings on Day 1 are shown in Table 1 and Figure 4. On Day 1, on a zero to 10 graphic rating scale, using a paired *t*-test, VR significantly reduced children's “worst pain” ratings during burn wound cleaning procedures in the ICU. On Day 1, worst pain during No VR = 8.52 (SD = 1.75) vs. during Yes VR = 5.10 (SD = 3.27), *t*_(47)_ = 7.11, *p* < 0.001, SD = 3.33, CI = 2.45–4.38, Cohen's d = 1.03, indicating a large effect size.

**Table 1:** Means (Standard Deviation) in “No-VR” condition vs. “Yes-VR” condition.

	**No-VR mean**	**Yes- VR**	***t* (df)**	***p-*value (Sig 2- tailed)**	**Confidence interval**	**Cohen's d effect size**	**Mean Diff**.	***SD* difference**
	**Mean (*SD*)**	**Mean (*SD*)**						
Worst pain	8.52	5.10	7.11	<0.001	2.45 to 3.33	1.03	3.42	3.33
	(1.75)	(3.27)	(47)			large		
Time spent thinking about pain	6.04	2.47	5.94	<0.001	1.86 to 3.76	0.87 large	2.81	3.24
	(3.41)	(3.37)	(46)			effect size		
Pain	6.40	3.47	5.49	<0.001	1.86 to 4.01	0.82 large	2.93	3.58
Unpleasant ness	3.51	(3.37)	(44)			effect size		
Fun	4.81	6.68	2.01	0.051 NS	3.75 to 0.004	0.29 small	1.87	6.39
	(3.93)	(3.86)	(46)			effect size		
Satisfaction with pain management	5.22	8.04	4.72	<0.001	1.59 to 4.07	0.99 large	2.83	2.87
	(3.34)	(2.33)	(22)			effect size		

*All 48 patients (44 children from developing countries and also 4 children from the USA)*.

### Descriptive Statistics About “Worst Pain” Ratings

On Day 1, the number of patients reporting excruciating pain (worst pain = 10) during wound care was 22 patients, which dropped to only five patients reporting excruciating pain (worst pain = 10) during Yes VR, and Cohen's d showed a strong effect size of VR analgesia. However, many of those patients with pain of 10 during No VR only dropped to 8 during VR (i.e., they dropped from excruciating pain during No VR down to severe pain during VR, but still reported severe pain during adjunctive VR).

*On Day 1, 40% of the 48 patients still reported pain of 7 or higher (severe to excruciating) during VR, despite receiving powerful traditional pharmacologic pain medications combined with immersive virtual reality*.

On average, patients spent mean = 16.56 min of wound care during No VR vs. 12.89 min during VR, *t*_(44)_ = 2.47, *p* < 0.05, SD = 9.97, CI =0.67–6.66, e.g., patients could not use VR while having their faces or heads cleaned. On Day 1, 14 of the 48 patients spent exactly the same amount of time during No VR (13.21 min) and during VR (13.21 min). These 14 patients also reported large and statistically significant reductions in pain during VR, worst pain during No VR = 8.50 (SD = 1.83), VR = 4.43 (SD = 3.08), *t*_(13)_ = 4.56, *p* < 0.005, SD = 3.34, CI = 2.14–6.00.

The mean number of days that patients rated their pain during Yes VR vs. during No VR was 4 study days. Collapsed across days, VR significantly reduced worst pain: Worst pain during “No VR” (Mean = 7.09, SD = 2.10) vs. worst pain during “Yes virtual reality” (Mean = 4.29, SD = 2.55), *t*_(47)_ = 7.32, *p* < 0.001, SD = 2.65, CI = 2.01–3.57, Cohen's d = 1.06, large effect size.

Consistent with the prediction that VR would continue to reduce pain when used day after day, a one-way within-subjects ANOVA comparing worst pain during “No VR” minus worst pain during “Yes VR” difference scores for days 1–7 showed no significant difference in the size of the VR analgesia effect over days 1–7, Wilks' Lamda *F*_(4, 6)_ = 1.50, *p* = 0.36, NS.

In exploratory analyses, patients scoring in the upper quartile on the children's pain catastrophizing score (PSC-C) in the current sample, showed significant VR analgesia. For patients scoring in the upper quartile on catastrophizing, mean worst pain during No VR = 7.00 (SD = 3.56), vs. VR = 2.86 (SD = 3.63), *t*_(6)_ = 2.80, *p* < 0.05, SD = 2.56, CI = 0.34 vs. 5.09. Patients scoring in the lower quartile in the current sample also showed significant VR analgesia, mean worst pain ratings during No VR = 6.00 (SD = 4.04), and during VR = 2.86 (SD = 2.85), *t*_(6)_ = 4.01, *p* < 0.01, SD = 1.03, CI = 1.61–6.67.

### Test of Secondary Hypotheses

As shown in Table 1 and Figure 4, on secondary GRS measures, on Day 1, pediatric burn patients reported large and significant reductions in pain on secondary measures of “pain unpleasantness” and “time spent thinking about pain during wound care.” Although children reported having 27% more fun during VR, the increase in fun on Day 1 was not statistically significant in the paired *t*-test. The children were significantly more satisfied with their pain management during VR, on average. Patients reported only a moderate illusion of “being there” inside the 3D computer generated world as if it was a place they visited. VR nausea was nearly zero (<1 on a 10 point scale).

The current study included 48 pediatric patients total. As shown in Table 2, in an exploratory analysis, to see if children from developing countries show VR analgesia, the subset (sub-analysis) of 44 patients from developing Latin American countries were analyzed separately from the four patients from the United States. As predicted, children from developing countries showed significant reductions in worst pain during VR, as well as significant reductions in pain unpleasantness (the emotional component of pain) and significant reductions in time spent thinking about pain during wound care (the cognitive component of pain). Encouragingly, analyzed separately, the four participants from the United States also showed the predicted patterns of large reductions of pain during VR.

**Table 2:** Means (Standard Deviation) in “No-VR” condition vs. “Yes-VR” condition.

	**No-VR**	**Yes- VR**	***t* (df)**	***p-*value (Sig 2- tailed)**	**Confidence interval**	**Cohen's d effect size**	**Mean Diff**.	***SD* difference**
	**Mean (*SD*)**	**Mean (*SD*)**						
Worst pain	8.43	5.20	6.65	<0.001	2.25 to 3.22	1.00	3.23	3.22
	(1.80)	(3.18)	(43)			large		
Time spent thinking about pain	5.86	3.33	5.32	<0.001	1.57 to 3.50	0.81 large	2.54	3.13
	(3.46)	(3.28)	(42)			effect size		
Pain unpleasant ness	6.26	3.57	5.17	<0.001	1.64 to 3.74	0.80 large	2.69	3.38
	(3.54)	(3.42)	(41)			effect size		
Fun	5.00	6.79	1.89	0.07 NS	3.70 to 0.12	0.29 small	1.79	6.21
	(3.85)	(3.76)	(42)			effect size		

*Sub-analysis of only the 44 children from developing Latin American countries (excluding the four children from the USA)*.

## Discussion

This pilot study provides preliminary evidence that immersive virtual reality can help reduce the pain of children with large severe burn wounds during burn wound cleaning in the Intensive Care Unit. Although using VR in the ICU hydrotank room was challenging and required creating custom equipment, in the current study, on Day 1, patients reported significant reductions in worst pain (pain intensity), children spent less time thinking about their pain during VR, children reported significant reductions in pain unpleasantness, and the children reported 27% higher ratings of fun during wound care during virtual reality. In addition, these pediatric patients were also significantly more satisfied with their pain management during virtual reality, they reported a moderate illusion of presence in VR (i.e., a moderately strong illusion of “being there” in the VR computer generated world during wound care), and VR nausea was nearly zero (<1 on a 10 point scale). Patients who received VR during more than 1 day of wound care continued to report the predicted pattern of reductions in worst pain during multiple wound care sessions. And patients with a tendency toward negative emotions and pessimistic beliefs about their ability to deal with the upcoming pain (i.e., patients in the upper quartile on catastrophizing), still benefitted from virtual reality distraction.

The reductions in worst pain ratings in the current study are similar to the pattern of VR analgesia reported in previous studies of 12 U.S. soldiers with combat-related burn injuries (TBSA of 21%) during wound care in their hospital beds. The soldiers spent 6 min in No VR vs. 6 min of wound care during VR (Maani et al., 2011a). In the current study the mean burn size was over 40%, the patients were all children, and the sample size was larger (*n* = 48 patients). Furthermore, in the current study, on average, patients spent over 12 min in VR and over 12 min in No VR, the wound care was conducted in the ICU instead of in the patients hospital beds, and the current study is the first to use a portable water-friendly VR system.

## Limitations

The demographics and characteristics of the participants of this pediatric pain study may limit generalization of findings of this study to other populations. Of interest is that 44 of the 48 patients were Spanish speaking patients from developing Latin American countries. As predicted, in an exploratory sub-analysis, the 44 children from developing Latin American countries showed statistically significant reductions in pain during VR. Encouragingly, analyzed separately, the four participants from the United States also showed the predicted patterns of large reductions of pain during VR. The VR system used in the current study was customized for use in the ICU hydrotank room, for patients with head and facial burns. Future randomized controlled trials research is needed to determine whether the current results replicate, and generalize to other VR systems.

Despite these limitations, the current study makes several important original contributions to the literature, and the results of the current study could have important implications for clinical practice: (a) this is the first study ever to attempt to use virtual reality during burn wound care in the intensive care unit, (b) the patients had unusually severe burn injuries much larger than burn injuries treated in any previous burn debridement VR analgesia study, (c) all of the patients were children, and 44 out of the 48 patients were Spanish speaking children from developing Latin American Countries, (d), the current study shows for the first time that children with large severe burns were generally able to play SnowWorld during severely painful medical procedures, and (e) playing SnowWorld in virtual reality significantly reduced worst pain ratings during wound care.

In the current study, a custom portable water-friendly VR system was used that did not have to physically contact the patient. The equipment was carefully cleaned with sterilizing cloths after each use, and the equipment was periodically swabbed/tested by the hospitals infection control team to test for the presence of any bacterial or viral pathogens. There was no problem with infection in the current study, using the custom VR system, which minimized or eliminated physical contact between the patient and the VR goggles. For patients with limited ability to wear VR helmets, modified VR systems that reduce contact surfaces (Hoffman et al., 2014) are highly recommended for use of VR during burn wound care for patients with severe unbandaged head and/or face burns. We also recommend discarding disposable foam liners that touch the patients face after each use. Burn patients are especially vulnerable to infections when unbandaged (during wound care), and VR equipment should be monitored by infection control, especially when used in the Intensive Care Unit.

## Conclusion

The results from the current pilot study support our hypothesis that immersive virtual reality can significantly reduce acute pain during burn wound care, even in pediatric patients with large severe burn wounds treated in the hydrotanks in the Intensive Care Unit. And VR continued to reduce pain when used day after day.

## Future Directions

Virtual reality (VR) may eventually prove to be “opioid sparing” during hospitalization (Kipping et al., 2012; McSherry et al., 2018). Additional research and development is needed on how to make VR analgesia more powerful (Wender et al., 2009), how to make pharmacologic pain medications more effective (McIntyre et al., 2016), and how to best combine pharmacologic pain medications and VR analgesia, to maximize total pain control. Development of more powerful new non-pharmacologic pain management techniques is a national and international priority (Keefe et al., 2018), and Virtual Reality has strong potential as a new direction for behavioral medicine (Keefe et al., 2012).

Fortunately, VR analgesia is not limited to severe burn patients, but could potentially be used for a wide range of painful medical procedures, and could be especially valuable for highly populated, lower income developing countries (4/5ths of the World's population), where large severe burns and other serious injuries are more common, and powerful pharmacologic analgesics are more scarce or unavailable. Additional research and development of VR analgesia is recommended.

## Data Availability

The datasets for this study will not be made publicly available because IRB restrictions.

## Ethics Statement

This research was conducted in accordance with the Declaration of the World Medical Association (www.wma.net). The studies were approved by the IRB from UTMB, and all participants provided written informed consent/assent in accordance with the Declaration of Helsinki.

## Author Contributions

All authors listed have made a substantial, direct and intellectual contribution to the work, and approved it for publication.

### Conflict of Interest Statement

All authors have completed the ICMJE uniform disclosure and declare support for the submitted work.
